# Recent Progress and Potential of G4 Ligands in Cancer Immunotherapy

**DOI:** 10.3390/molecules30081805

**Published:** 2025-04-17

**Authors:** Jiahui Lin, Zhu Gong, Yingyue Lu, Jiongheng Cai, Junjie Zhang, Jiaheng Tan, Zhishu Huang, Shuobin Chen

**Affiliations:** School of Pharmaceutical Sciences, Sun Yat-sen University, Guangzhou 510006, China; linjh99@mail2.sysu.edu.cn (J.L.); gongzh26@mail2.sysu.edu.cn (Z.G.); luyy79@mail2.sysu.edu.cn (Y.L.); caijh53@mail2.sysu.edu.cn (J.C.); zhangjj259@mail2.sysu.edu.cn (J.Z.); tanjiah@mail.sysu.edu.cn (J.T.); ceshzs@mail.sysu.edu.cn (Z.H.)

**Keywords:** G-quadruplex, cancer immunotherapy, anticancer agent

## Abstract

G-quadruplex (G4) structures are non-canonical nucleic acid conformations that play crucial roles in gene regulation, DNA replication, and telomere maintenance. Recent studies have highlighted G4 ligands as promising anticancer agents due to their ability to modulate oncogene expression and induce DNA damage. By stabilizing G4 structures, these ligands affect tumor progression. Additionally, they have been implicated in tumor immunity modulation, particularly through the activation and immunogenic cell death induction of the cyclic GMP–AMP synthase (cGAS)–stimulator of interferon genes (STING) signaling pathway. Moreover, their disruption of telomere maintenance and regulation of key oncogenes, such as *c-MYC* and *KRAS*, position them as candidates for immune-based therapeutic interventions. Despite their therapeutic potential, challenges remain in optimizing their clinical applications, particularly in patient stratification and elucidating their immunomodulatory effects. This review provides a comprehensive overview of the mechanisms through which G4 ligands influence tumor progression and immune regulation, highlighting their potential role in future cancer immunotherapy strategies.

## 1. Introduction

**Figure 1:**
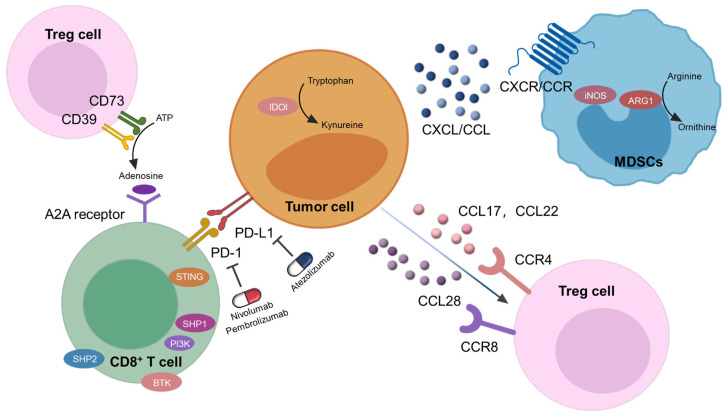
Key mechanisms of tumor immune escape.

The immune system is a highly sophisticated and precise defense mechanism that regulates immune surveillance and response. When cancer invades, the immune system recognizes tumor-associated antigens on the cell membrane, initiating specific humoral and cellular immune responses that are aimed at eliminating malignant cells. However, cancer cells exploit multiple immunosuppressive pathways to evade immune detection—a process known as tumor immune escape [1]. This evasion enables tumors to grow unchecked, often facilitated by the tumor microenvironment (TME), a complex ecosystem that supports tumor progression [2,3]. Key immune escape mechanisms include the upregulation of immune checkpoint proteins, such as PD-L1, which inhibits T cell activation [4,5,6], and the secretion of immunosuppressive molecules, like adenosine, further dampening the anti-tumor immune responses [7]. Additionally, the recruitment of regulatory T cells (Tregs) [8,9] and myeloid-derived suppressor cells (MDSCs) into the TME contributes significantly to immune suppression, reducing the effectiveness of anti-tumor responses (Figure 1) [10,11,12].

Cancer immunotherapy has emerged as a transformative approach that harnesses the immune system to target cancer cells. Unlike conventional therapies, such as chemotherapy and radiotherapy, immunotherapy provides enhanced specificity and fewer side effects. Immune checkpoint inhibitors, including the PD-L1 monoclonal antibody atezolizumab and PD-1 monoclonal antibodies nivolumab and pembrolizumab, have demonstrated significant clinical efficacy [13]. However, due to the complexity of the TME, current immune checkpoint inhibitors remain insufficient for complete tumor eradication, underscoring the need for novel therapeutic strategies. Recent advancements in drug development have identified promising molecular targets, such as lymphocyte activation gene 3 (LAG-3), T cell immunoreceptor with immunoglobulin and ITIM domain (TIGIT), and T cell immunoglobulin and mucin domain-3 (TIM-3). Additionally, combination therapies are advancing through clinical trials, further shaping the evolving landscape of cancer immunotherapy [14,15,16].

G-quadruplexes (G4s) are non-canonical secondary structures formed by the folding of guanine-rich nucleic acid sequences. These unique conformations play a pivotal role in tumor biology, particularly in regulating the TME. The stabilization and accumulation of G4 structures have been implicated in various oncogenic processes, including immune evasion. Emerging evidence suggest that G4 ligands modulate tumor immune escape through diverse mechanisms, presenting a promising avenue for therapeutic intervention. In light of these findings, this review explores the potential of G4 ligands in enhancing cancer immunotherapy.

## 2. Overview of G4 Structures and Biological Roles

**Figure 2:**
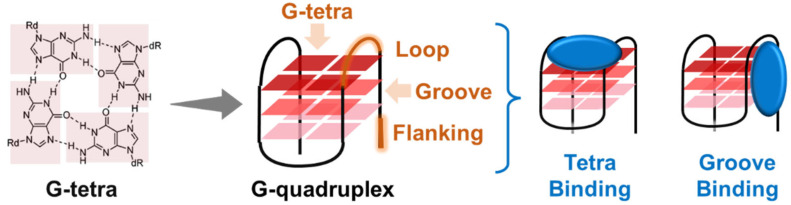
Schematic of G4 structures and small molecule binding site.

G4s are stable four-stranded helical structures formed within guanine-rich DNA and RNA sequences (Figure 2) [17]. These structures can assemble through either intramolecular or intermolecular interactions. The fundamental unit of a G4 is the G-tetrad, in which four guanine residues form a cyclic, Hoogsteen hydrogen-bonded square planar arrangement [18]. Two or more G-tetrads stack to form the G4 structure, which is further stabilized by monovalent cations, such as Na^+^ and K^+^ [19]. G4s can adopt distinct topological conformations, including parallel, antiparallel, and hybrid, depending on ionic conditions and sequence context [20]. In addition to the core conformation, the loops connecting the G-tetrads exhibit diverse structural variations, such as propeller, V-shaped, lateral, diagonal, and snapback loops [21]. These structural elements, including grooves, tetrads, and loop configurations, play crucial roles in molecular recognition, allowing various biomolecules and small molecules to selectively interact with G4s, thereby influencing their biological functions.

Putative G4 sequences (PQSs) are widely distributed in key genomic regions, including telomeres, ribosomal DNA, promoter regions, and mRNA untranslated regions (UTRs) [22]. Computational analyses have identified approximately 370,000 PQSs in the human genome [23]. Furthermore, experimental evidence indicates that G4s can form in sequences beyond the canonical PQS motifs, including those with longer loops or fewer than three guanines per G-tract [24,25]. Additionally, G4 formation has been detected in sequences that deviate from the PQS consensus [26,27]. High-throughput sequencing approaches have revealed over 700,000 potential DNA PQS regions [28] and approximately 13,000 RNA PQS regions within the human genome [29], highlighting the widespread presence of G4s. Their distribution underscores their essential roles in cellular processes, including telomere maintenance, ribosome biosynthesis, DNA replication, transcription, and translation [30]. Notably, PQSs are highly enriched in oncogenes, suggesting their involvement in tumorigenesis. However, G4-ChIP analyses in living cells have estimated that only ~1.5% of PQSs actually form stable G4 structures [31], indicating that the functional impact of G4s is more limited than their theoretical presence suggests.

G4s are more prone to folding in cancer cells, drawing considerable attention to their biological roles in tumorigenesis. Studies over the past decades have demonstrated that G4 formation can lead to gene silencing [32] and DNA damage at G4 sites [33], affecting oncogenes, telomeres, and mitochondrial DNA (mtDNA). As a result, G4s have emerged as promising targets for anticancer therapies. The biological functions of G4 structures are particularly well-understood in two major contexts: telomeres and promoter regions. Telomeres, characterized by long G-rich repetitive sequences (TTAGGG), can form multimeric G4 structures [34], which play a crucial role in telomere function regulation [22]. For instance, the folding of telomeric G4s can inhibit telomerase activity, leading to telomere shortening and cellular senescence [35]. Moreover, the formation of telomeric G4s can destabilize the protective telomeric structure, triggering DNA damage responses and promoting apoptosis [36]. Notably, telomeric G4s exhibit structural heterogeneity and may exert complex regulatory effects through cooperative interactions [37].

Another well-characterized G4 structure is found in the *c-MYC* oncogene. The human *c-MYC* gene contains a G-rich sequence in its P1 promoter region at the NHE III site [38]. This sequence, known as Pu27, forms a stable parallel G4 conformation and plays a key role in regulating *c-MYC* transcription [39]. G4 formation in this region interferes with RNA polymerase activity and influences the binding of transcription factors, such as SP1 and CNBP, typically leading to transcriptional repression [40]. Given that *c-MYC* is a critical oncogene, the *c-MYC* G4 has been widely explored as a therapeutic target. Numerous small-molecule ligands have been developed to selectively bind and stabilize this G4 structure, with some advancing into clinical trials. Interestingly, emerging evidence suggests that under certain conditions, *c-MYC* G4 folding may facilitate gene transcription, and small molecules may regulate transcriptional outcomes by modulating specific protein interactions [41]. Regardless of the precise mechanism, G4 structures are integral to tumor biology and represent promising molecular targets for therapeutic intervention.

## 3. Overview of G4 Ligands as Anticancer Agents

### 3.1. Representative G4 Ligands

**Figure 3:**
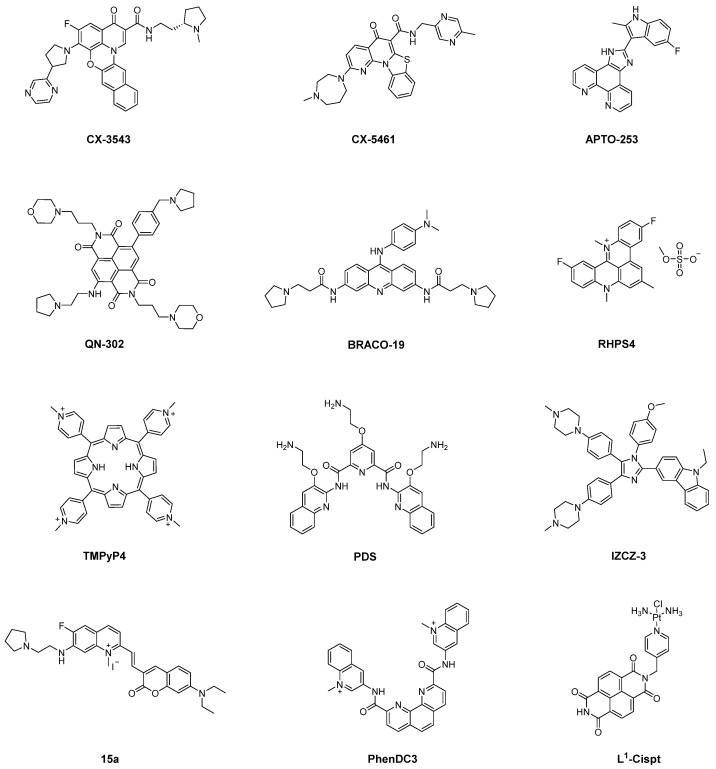
Representative G4 ligands.

Given the critical role of G4 structures in tumor biology, their stabilization or disruption can significantly influence tumor proliferation and oncogene expression. Small-molecule ligands that selectively target G4 structures have demonstrated promising efficacy in inhibiting cancer cell growth (Figure 2) [42]. Consequently, developing G4 ligands with high specificity and stability has substantial pharmaceutical significance. Several such ligands have progressed into clinical trials as potential anticancer drug candidates (Figure 3).

G4LDB is one of the first databases dedicated to G4 ligands. The latest version, G4LDB 3.0, includes more than 4800 G4 ligands [43,44]. These G4 ligands typically share key structural features, including an aromatic scaffold, hydrogen bond donors or acceptors, and electropositive groups. These characteristics enable their interaction with G4 structures via π-π stacking, hydrogen bonding, and electrostatic interactions [42]. One of the earliest and most studied G4 ligands is the fluoroquinolone derivative **CX-3543** (**Quarfloxin**), the first G4 ligand to enter clinical trials. **CX-3543** modulates tumor proliferation by stabilizing the ribosome DNA (rDNA) G4–nucleolin complex [45,46]. Although it exhibited a favorable safety profile in clinical trials (NCT00955786), its efficacy in patients fell short of expectations. Building on **CX-3543**, **CX-5461** (**Pidnarulex**) was designed to stabilize DNA G4 structures and promote DNA damage in cancer cells. This ligand has currently finished Phase I clinical trials (NCT02719977), reinforcing the therapeutic potential of G4 ligands in cancer treatment [45,47].

Additionally, two other G4 ligands, **APTO-253** and **QN-302**, have also entered clinical evaluation. **APTO-253**, a 2-indolyl imidazole [4,5-*d*] phenanthroline derivative [48], is the third G4 ligand to reach clinical trials. It stabilizes G4 structures and downregulates *c-MYC* expression in acute myeloid leukemia (AML) cells [49]. However, its Phase I clinical trial (NCT02267863) was terminated due to formulation issues. **QN-302**, a naphthalene diimide derivative (previously known as **SOP1812**), is the fourth G4 ligand to enter clinical trials. Its four amino side chains enable interactions with G4 grooves, thereby inhibiting the binding of transcription factors to oncogenes and suppressing RNA polymerase activity, ultimately downregulating over 80 genes [50]. In 2023, the FDA granted **QN-302** orphan drug designation, and it was approved for a Phase I clinical trial (NCT06086522).

Beyond these clinical candidates, numerous G4 ligands have been extensively studied for their ability to modulate G4 structures. Acridine-based ligands are among the most significant classes of G4 ligands. For instance, **BRACO-19**, a 3,6,9-trisubstituted acridine exhibits strong binding affinity and stability toward telomeric G4s [51,52,53]. Another well-known acridine derivative, **RHPS4**, targets both telomeric and telomerase-associated G4s, inducing telomere dysfunction [54,55]. Studies suggest that **RHPS4** may also bind mtDNA G4s, thereby inhibiting mtDNA transcription at low concentrations [56].

Porphyrin-based ligands, such as **TMPyP4**, and **telomestatin** derivatives also represent critical G4 ligands. These compounds, featuring planar aromatic ring systems, primarily bind G4 structures through π–π stacking interactions with the G-quartet. **TMPyP4**, a well-known cationic porphyrin, exhibits high stability toward telomeric and oncogenic G4s [57,58]. However, strong interactions were also observed between **TMPyP4** and duplex DNA. **Telomestatin**, a natural product derived from Streptomyces anulatus, selectively binds and stabilizes telomeric G4s with greater specificity than **TMPyP4** [59].

In contrast, certain G4 ligands lack large planar aromatic systems, reducing their likelihood of intercalating into double-stranded DNA. One example is the G4-selective ligand, **pyridostatin** (**PDS**). It is widely proved that **PDS** and its derivatives exhibited high specificity for G4s [60]. Notably, **PDS** derivatives with different kinds of side chains enabled enhanced selectivity toward different kinds of G4s [61]. Recently, **MitoPDS** was developed from **PDS**, which accumulates in mitochondria and selectively binds mtDNA G4s, subsequentially activating glycolysis-related genes [62]. Additionally, coumarin–quinolinium derivative **15a** and bisquinolinium derivative **PhenDC3** have been also demonstrated strong G4-binding specificity [63,64]. We identified triaryl-substituted imidazoles as a promising class of G4-selective ligands [65]. Ligands based on this scaffold exhibit distinct selectivity toward different G4s. **IZNP-1** preferentially binds telomeric G4s. The K_D_ values for multimeric telomeric G-quadruplexes were around 10.0 μM, respectively, while no significant bindings were observed with regard to other G4s [66] and **IZCZ-3**, a novel four-leaf clover-like ligand that selectively targets the *c-MYC* promoter. Fluorescence titration results show that the emission peak of **IZCZ-3** shifts and exhibits a significant fluorescence enhancement with the gradual addition of the parallel *c-MYC* G4 pu22. In contrast, the hybrid telomeric G4 htg22 also leads to a red shift, but the fluorescence signal shows only a slight enhancement even of the high concentration [67]. By utilizing a PCR-stop assay, a study demonstrates that **telomestatin** and **TMPyP4** are telomeric and *c-MYC* equipotent ligands, while **BRACO-19** presented a fivefold preference for the *c-MYC* sequence [68].

Metal-based complexes represent a significant class of G4 ligands, with their positively charged metal centers markedly enhancing G4 binding affinity. Additionally, the unique spatial configurations formed through coordination confer distinct selectivity to these compounds. For instance, nickel complexes exhibiting zinc finger-like chiral supramolecular structures preferentially bind to higher-order G4 over single G4 [69]. Similarly, platinum-based G4 ligands have been developed to improve G4 specificity. A notable example is a novel class of G4-binding organic platinum hybrids, **L^1^-Cispt** and **L^1^-Transpt**, which were designed to enhance selective G4 recognition. Structural analyses revealed that **L^1^-Transpt** disrupts specific base pairs within the MYT1L G4 sequence, enabling the platinum center to covalently bind the N7 position of guanine, thereby stabilizing the complex through π–π stacking interactions [70,71].

Notably, several of the ligands mentioned above exhibit selectivity toward specific G4 structures, particularly telomeric G4s and *c-MYC* G4s. These two types of G4s differ significantly in their structural features, including their folding topologies, loop arrangements, and flanking sequences at both the 3′ and 5′ termini. Consequently, ligands equipped with functional groups that specifically interact with these structural elements, such as the loops and flanking regions, may preferentially recognize certain G4 conformations over other nucleic acid structures [72]. However, because different G4s were similar, the development of selective G4 ligands towards individual G4s was still challenging.

### 3.2. Pharmacologic Effect of G4 Ligands

#### 3.2.1. Inducing Telomere Shortening

Telomeric G4 structures represent a crucial therapeutic target in cancer treatment. Since telomerase, the enzyme responsible for telomere maintenance, has long been difficult to regulate pharmacologically, targeting telomeric G4 structures has emerged as a promising alternative strategy [73,74,75]. Numerous G4 ligands have been developed to selectively bind and stabilize telomeric G4s, thereby impairing telomere function. Research further demonstrates that G4 ligands inhibit telomerase binding to telomeric DNA by stabilizing G4 structures, thereby preventing telomere elongation [76]. By stabilizing telomeric G4s, G4 ligands suppress telomerase activity, leading to telomere shortening and the activation of DNA damage response through ataxia-telangiectasia mutated/ATM and Rad3-related (ATM/ATR) signaling pathways.

Among them, **BRACO-19** has been extensively studied, providing critical insights into the biological consequences of telomeric G4 stabilization [53,77]. Studies indicate that **BRACO-19** induces telomere dysfunction in cancer cells through two primary mechanisms. First, it inhibits telomerase activity, leading to progressive telomere shortening and eventual cellular senescence [78]. Second, **BRACO-19** interferes with the function of the telomeric 3′ overhang, disrupting the binding of sheltering protein telomeric repeat-binding factor 2 (TRF2) and human protection of telomeres 1 (hPOT1) at telomere ends. This interference promotes telomere uncapping, subsequently activating the DNA damage response (DDR) [52,79,80].

Similarly, **RHPS4**, another well-characterized G4 ligand, stabilizes telomeric G4 DNA and disrupts critical telomere processing events. This impairment triggers telomere dysfunction and induces replication stress responses [54,81,82]. Specifically, **RHPS4** significantly reduces the incorporation of thymidine analogs, such as BrdU at telomeric regions, indicating the perturbation of telomere replication dynamics. Moreover, **RHPS4** affects the dynamic binding of TRF1, TRF2, and hPOT1, leading to telomere disorganization [83,84]. This disruption promotes the formation of telomeric DNA dimers, resulting in chromosomal aberrations and the subsequent activation of the ATM/ATR signaling pathways [85].

#### 3.2.2. Inducing DNA Damage

One of the key mechanisms by which G4 ligands exert their anticancer effects is by inducing double-strand breaks (DSBs) in non-telomeric DNA regions, thereby promoting genomic instability. G4 structures preferentially form near replication forks, where they can act as physical barriers to DNA replication [86]. Under normal conditions, cells rely on intrinsic DNA repair mechanisms to resolve such replication-associated damage and maintain genomic integrity [87]. However, studies have demonstrated that G4 ligands stabilize these structures, increasing G4 accumulation at replication forks and thereby exacerbating replication stress, ultimately leading to DSBs [88].

By promoting DSB accumulation, G4 ligands activate DDR pathways. For instance, **PDS** interacts with non-telomeric DNA G4s, inducing DNA damage linked to both replication and transcriptional stress. This activation triggers DNA damage checkpoints, leading to cell cycle arrest and the subsequent inhibition of cancer cell proliferation [89]. Moreover, treatment with the G4 ligand **20A** in HeLa cells significantly enhanced DDR signaling. The activation of key DDR pathways, including ataxia–telangiectasia mutated/checkpoint kinase 2 (ATM/CHK2) and ATR/CHK1, ultimately drives cellular senescence and apoptosis in cancer cells [90]. These findings suggest that cancer cells with impaired DNA repair mechanisms may exhibit heightened sensitivity to G4 ligands. For example, cancer cells harboring homologous recombination repair (HRR) defects, such as BRCA1/2-deficient breast cancer cells, have been demonstrated increased susceptibility to G4 ligand-induced DNA damage [88].

The DNA damage induced by G4 ligands may also be linked to R-loop formation, with specific cellular responses dependent on the chemical properties of the ligand. R-loops are DNA-RNA hybrids that structurally resemble G4s. Unregulated R-loop formation can result in DSBs, genomic instability, and cell death. G4 ligands, such as **PDS**, were identified to induce the formation of both G4 structures and R-loops in the close chromatin domains of HeLa cells. Furthermore, R-loop structures were significantly extended in G4-rich genomic regions, suggesting that G4 ligands can stabilize both G4 and R-loops within chromatin. Subsequent transfection experiments and cytotoxicity assays confirmed that R-loop formation plays a critical role in the DNA damage and cytotoxic effects induced by G4 ligands, including in BRCA2-deficient cells. Additionally, R-loop stabilization mediated an increase in micronuclei formation in **PDS**-treated cells, further highlighting the physiological role of R-loops in G4 ligand-induced genomic instability [91].

Beyond nuclear DNA, cancer cells exhibit a higher propensity for G4 formation within mtDNA than normal cells [62]. The stabilization of these mitochondrial G4 structures by G4 ligands can lead to mtDNA instability and alterations in cellular metabolism. For instance, the highly selective mtDNA G4 ligand **MitoPDS** has been shown to induce mtDNA instability and upregulate glycolysis. Similarly, other mtDNA G4 ligands, such as **BKN-1**, have been found to cause mtDNA damage [92]. However, whether mtDNA damage directly promotes cell death remains uncertain, as **MitoPDS**, despite its high selectivity, did not exhibit cytotoxicity at effective concentrations.

#### 3.2.3. Modulating Oncogene Expression

Oncogenes play a critical role in the progress of tumorigenesis. The inhibition of oncogene expression has emerged as a promising therapeutic method in cancer treatment. Notably, studies have illuminated the role of G-quadruplex (G4) structures in the regulation of these oncogenes, particularly in their promoter regions and untranslated regions (UTRs). The formation of G4s in these key regulatory regions, such as those found in *c-MYC*, *KRAS*, and others, has been associated with reduced gene expression, further suggesting that targeting these G4 structures could serve as an effective approach for anti-cancer therapies [93,94].

Among the various oncogene G4s, the *c-MYC* G4 is most extensively studied. *c-MYC* is a pivotal oncogene, whose aberrant activation is strongly linked to enhanced cancer cell proliferation, metabolic reprogramming, and the promotion of tumorigenesis. The *c-MYC* promoter region contains a G-rich sequence capable of forming a stable G4 structure, which directly modulates its transcription. Numerous small-molecule ligands have been developed to specifically target and stabilize the *c-MYC* G4, thereby inhibiting its transcription. One notable example is the clinical trial agent **APTO-253**, which effectively inhibits *c-MYC* expression in various cancer cell lines, including those from colon cancer, leukemia, non-small-cell lung cancer, renal cancer, and prostate cancer [49,93]. **APTO-253**’s potent anti-proliferative effects underscore the therapeutic potential of G4 ligands in disrupting oncogene expression and impeding cancer cell growth.

In addition to *c-MYC*, *KRAS* is another oncogene with significant clinical relevance. *KRAS* mutations are prevalent in a wide range of cancers, including pancreatic cancer (up to 90%), colorectal cancer (50%), and lung adenocarcinoma (30%) [94]. These mutations often result in the overexpression of *KRAS*, contributing to poor prognosis and resistance to conventional therapies. Similar to *c-MYC*, *KRAS* contains G-rich sequences in its promoter region that can fold into G4 structures, influencing its transcriptional regulation. The *KRAS* DNA G4 has been identified as a key modulator of gene expression, and stabilizing or destabilizing this structure can significantly impact cancer progression. For instance, a study demonstrated that alkaloids, such as **berberine** and **coptisine** bind to the *KRAS* DNA G4 in the promoter region, effectively inhibiting *KRAS* transcription in non-small-cell lung cancer cells (H460 and A549) [95].

In addition to the promoter G4s, G4s in the 5′-UTR of the gene have also been implicated in the regulation of translation. Interestingly, G4 also occurs in the 5′-UTR of *KRAS* mRNA. A *KRAS* RNA G4 ligand **15a** demonstrated its ability to effectively inhibit *KRAS* protein expression in pancreatic cancer cells (MIA PaCa-2 and PANC-1) without altering the mRNA levels, suggesting that **15a** acts as a *KRAS* translational suppressor [63]. This highlights how G4 ligands can modulate oncogene expression at both the transcriptional and translational levels, providing a dual mechanism for therapeutic intervention in cancer.

Overall, the development of G4 ligands that specifically target oncogene-associated G4 structures has emerged as a promising strategy for cancer therapy. By modulating gene expression through stabilizing G4s, these ligands can disrupt key oncogenic pathways that drive tumor progression.

Some G4 ligands that destabilize G4 structures exhibit unfolding activities similar to those of helicases, such as **TMPyP4** and **TAP1**. However, the potential of these chemicals as surrogates for helicases are yet to be shown, given that no in-depth cellular investigations have been performed [96,97].

## **4.** Potential **of G4 Ligands in Cancer Immunotherapy**

Small-molecule ligands targeting nucleic acids have emerged as key agents in inducing DNA damage and activating endogenous immune pathways, further advancing their potential in cancer immunotherapy. Recent studies indicate that numerous clinically relevant chemotherapy drugs possess tumor immunomodulatory functions. For instance, the widely used DNA-damaging agent cisplatin has been shown to activate the cGAS-STING pathway, promoting T cell accumulation within the TME. This activation leads to the upregulation of immunogenic markers, such as calreticulin (CRT), major histocompatibility complex class Ⅰ (MHC I) molecules, and molecules involved in antigen processing and presentation, including transporter associated with antigen processing 1/2 (Tap1/2) and lysosomal associated membrane protein 2/10 (LAMP2/10), thereby enhancing the immunogenicity of cancer cells [98]. The ongoing clinical trials investigating combination therapies that target the cGAS-STING pathway highlight the growing significance of DNA damage induction in shaping effective tumor immunotherapy strategies [99].

**Figure 4:**
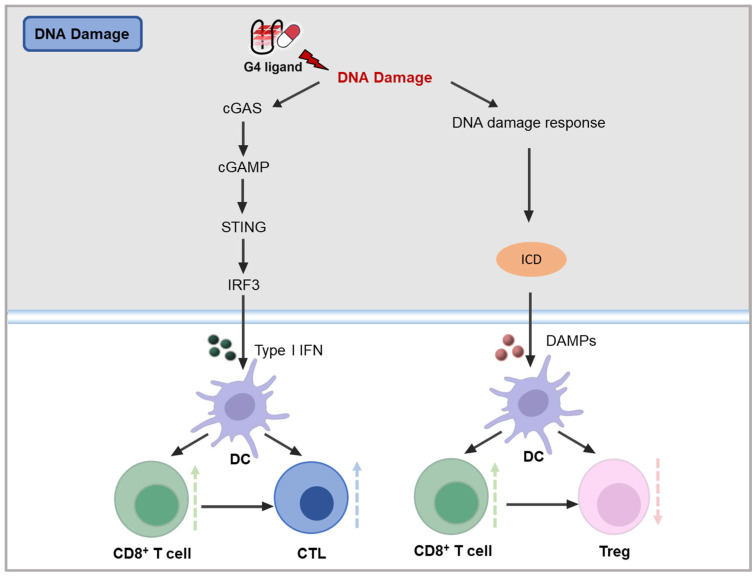
Potential mechanisms of DNA damage pathway on tumor immunity [100,101,102,103,104,105,106].

The cGAS-STING pathway is a pivotal component of the intracellular immune response to cytosolic DNA, playing a critical role in how cancer cells react to DNA damage by activating immune responses. This pathway is initiated when cGAS recognizes cytosolic double-stranded DNA and catalyzes the synthesis of cGAMP from ATP and GTP [100]. As a second messenger, cGAMP binds and activates the STING [101]. Once activated, STING translocates from the endoplasmic reticulum to the signaling compartment, where it facilitates the phosphorylation of the transcription factor interferon regulatory factor 3 (IRF3) by TANK-binding kinase 1 (TBK1) [102]. Phosphorylated IRF3 then translocates to the nucleus, where it interacts with other transcription factors to induce the production of type I interferons (IFN-I) [103,104]. IFN-I, in turn, promotes dendritic cells (DCs) to cross-present antigens to CD8^+^ T cells, thereby enhancing tumor-specific cytotoxic T lymphocytes (CTLs) [105]. This cascade of events exemplifies the process of immunogenic cell death (ICD), wherein cancer cells shift from being non-immunogenic to immunogenic after external stimuli-induced cell death. ICD stimulates a robust anti-tumor immune response by promoting DC maturation, antigen presentation, T cell priming, and recruitment, ultimately boosting the cytotoxicity of CTLs [106]. The feasibility of this approach has been demonstrated in clinical settings, particularly through liposomal oxaliplatin treatment (Figure 4) [107].

The potential of G4 ligands in cancer immunotherapy lies in their ability to induce DNA damage, a process that is integral to triggering the cGAS-STING pathway and enhancing immunogenicity. G4 ligands, by binding to G4 structures within key genomic regions, can promote DNA strand breaks and structural destabilization, resulting in the activation of DDR pathways. This damage can stimulate immune responses by initiating the production of immunogenic molecules and triggering ICD (Figure 4). Several classes of G4 ligands, including those that stabilize or destabilize G4 structures, have shown promise in modulating tumor immune responses. These ligands not only affect tumor proliferation and gene expression but may also serve as potent activators of the immune system, positioning them as promising agents for enhancing cancer immunotherapy strategies. This review now explores the immunomodulatory effects of diverse G4 ligands within the context of cancer immunotherapy.

### 4.1. DNA Damage Inducing G4 Ligands

G4 ligands are potent inducers of DSBs by stabilizing G4 structures, which play a key role in their ability to activate immune responses. It has demonstrated that well-characterized G4 ligands, such as **PDS** and **PhenDC3**, can induce DSBs at non-toxic concentrations, leading to the release of DNA fragments into the cytoplasm. This process activates the cGAS-STING pathway, triggering the secretion of pro-inflammatory cytokines, including IFN-β [108]. This finding further corroborates the notion that G4 ligands, akin to traditional chemotherapeutic agents, can induce DNA damage, activate the DDR, and activate tumor-associated immune signaling pathways. Subsequent studies have shown that a variety of G4 structures can induce similar effects. For instance, platinum (II) triphenylamine complexes, **Pt1** and **Pt2**, also activate the cGAS-STING pathway and release pro-inflammatory cytokines at toxic concentrations [109], while platinum (II) ligand **CP** induces both the cGAS-STING pathway and pyroptosis-related AIM2-ASC signaling, leading to ICD in MDA-MB-231 cells above the toxic concentration [110].

Beyond inducing DSBs in the nucleus, G4 ligands have also been shown to cause mtDNA damage, further contributing to immune activation. Research has indicated that the triphenylamine-based ligand **A6** targets and stabilizes G4 structures in mtDNA, causing mtDNA damage and activating the cGAS-STING pathway in 4T1 cells. However, the concentration required for immune activation is notably higher than the toxic concentration, suggesting that the primary anticancer effect of **A6** may be attributed to its direct cytotoxicity rather than immune modulation [111].

These findings suggest that G4 ligands, by inducing DNA damage in both nuclear and mtDNA and promoting genomic instability, can activate the cGAS-STING pathway, offering promising potential for cancer immunotherapy. Despite these promising results, research on the relationship between G4 ligands and tumor immunity remains limited. Most studies primarily focus on monitoring the activation of the cGAS-STING pathway (including cGAS, STING), ICD-inducing damage-associated molecular patterns (DAMPs), such as calreticulin (CRT), ATP, and high mobility group box 1 (HMGB1), and downstream effects, such as DC maturation and CD8^+^ T cell infiltration within the TME. However, there remains a notable gap in research exploring the broader immune-related signaling pathways and the comprehensive immune response triggered by G4 ligands. Most studies focus on the activation of the cGAS-STING pathway or ICD at concentrations above the cytotoxic IC_50_. Thus, the full potential of G4 ligands inducing tumor immunity through DNA damage pathways warrants further investigation and a more thorough exploration of the immune responses at varying concentrations.

### 4.2. Telomere Shortening Inducing G4 Ligands

Emerging research highlights a critical link between telomeric DNA damage and tumor immune responses [112]. Under pathological conditions, such as oxidative stress and chronic inflammation, telomeres undergo structural destabilization, resulting in the release of telomeric DNA damage fragments [113,114]. These fragments can activate ICD similar to the effect induced by DSBs from G4 ligands [115,116].

**Figure 5:**
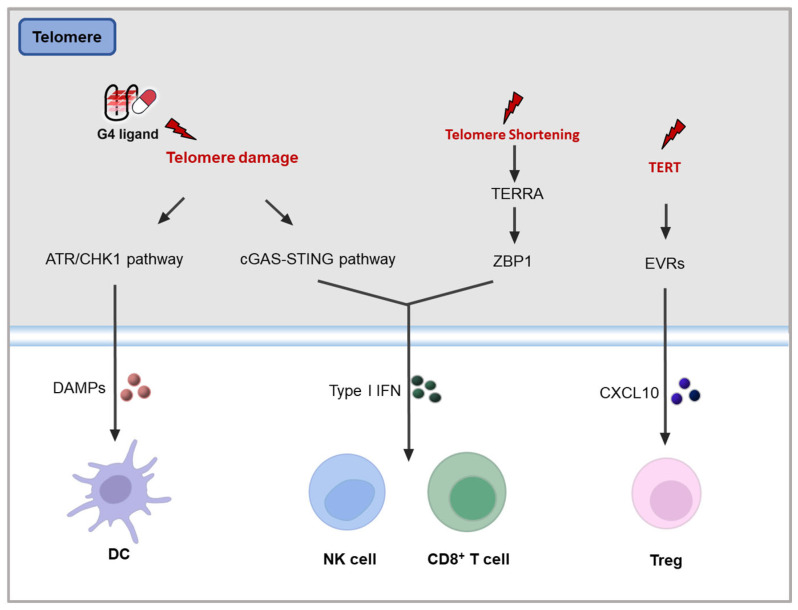
Potential mechanisms of telomere pathway on tumor immunity [117,118,120].

Research has shown that **PI-2**, a G4 ligand, can inhibit the proliferation and migration of both human and mouse triple-negative breast cancer (TNBC) cells, while also triggering ICD. The associated signaling pathways have been clearly identified [117]. This finding strongly supports the hypothesis presented earlier that G4 small-molecule ligands can induce ICD by causing telomeric damage. Specifically, **PI-2** stabilizes telomeric G4 structures, activates the ATR/CHK1 pathway, and subsequently, leads to the exposure of CRT and the release of HMGB1 and ATP, thereby activating anti-tumor immunity (Figure 5) [117].

Furthermore, studies have indicated that telomeric DNA fragments, resulting from telomere damage, can stimulate anti-tumor immune responses through the activation of the cGAS-STING pathway. A prominent example is the pharmacological telomerase inhibitor **6-thio-2′-deoxyguanosine** (**6-thio-dG**), which induces telomere dysfunction by targeting telomerase-positive cancer cells [112]. This disruption leads to the accumulation of cytoplasmic telomeric DNA fragments that are recognized by DCs, thereby activating the cGAS-STING pathway, promoting IFN-I production, and enhancing T cell-mediated anti-tumor immunity (Figure 5).

G4 ligands can inhibit telomerase activity and disrupt the protective structure of telomeres by binding to telomeric G4s, leading to telomeric damage [54,77,81,82]. Given that G4 ligands targeting telomeres can induce telomeric damage, it is plausible that these ligands might also activate tumor immune responses through the cGAS-STING pathway. However, there are limited reports exploring such functions of G4 ligands.

Additionally, extremely short telomeres can trigger tumor immunity through mitochondrion-related pathways [118]. Research has demonstrated that when telomeres shorten to a certain extent, they synthesize a specific non-coding RNA known as telomeric repeat-containing RNA (TERRA). TERRA is transcribed from repeated sequences of telomeric DNA and has unique biological functions. It can localize to the outer surface of mitochondria, activating two key immune sensors: Z-DNA binding protein 1 (ZBP1) and mitochondrial antiviral signaling protein (MAVS). This process mimics the activation of nonspecific immunity, similar to viral RNA recognition. Upon TERRA presence, ZBP1 undergoes conformational changes, triggering downstream IFN signaling pathways, while MAVS enhances the inflammatory immune response. Consequently, inflammatory factors and immune cells target and destroy cells with extremely short telomeres and genomic instability, preventing their transformation into cancer cells (Figure 5). Recent studies have shown that the small molecule **HIT17** can effectively stabilize TERRA G4 [119]. The stabilization of TERRA G4 in vitro induces the dissociation of TRF2 from telomeres, activating an ATM-dependent DNA damage response, cell cycle arrest, proliferation blockade, and apoptotic death in multiple myeloma (MM) cell lines. This suggests that TERRA G4 stabilizers have potential in activating tumor immune responses.

Moreover, telomerase inhibitors may reverse the immunosuppressive TME by inhibiting telomerase activity, thus activating anti-tumor immunity [120]. Telomerase, an essential ribonucleoprotein reverse transcriptase, adds telomere DNA to the ends of eukaryotic chromosomes [121]. In normal human tissues, telomerase activity is suppressed, but in cancer cells, telomerase is reactivated, enabling unlimited proliferation [122,123]. Telomerase reverse transcriptase (TERT) plays a critical role in tumorigenesis. Studies have shown that TERT can activate endogenous retroviruses (ERVs), which produce double-stranded RNA (dsRNA) through divergent transcription [120]. This dsRNA activates interferon signaling in cancer cells, promoting the expression of chemokines and resulting in the infiltration of suppressor T cells, which contribute to the formation of an immunosuppressive TME (Figure 5). In this context, telomerase inhibitors could potentially enhance tumor immunity by directly modulating the TME. However, few studies have reported G4 ligands with telomerase inhibitory activity showing such functions.

Numerous G4 ligands have been identified that can stabilize telomeric G4s [124], which exhibit stronger selectivity and stability for telomeres than **PI-2** and have been thoroughly validated for their ability to induce telomeric damage. However, the potential applications of most telomeric G4 ligands in tumor immunity have not been fully explored. Findings related to **PI-2** provide evidence that telomeric ligands may induce ICD through DDR pathways. It is crucial to recognize that telomere-related tumor immune regulatory events extend beyond merely DNA damage pathways, such as the TERRA pathway. Importantly, telomeric G4 ligands may also directly interact with surrounding immune cells, influencing tumor immunity by modulating telomere functions within these cells. Therefore, telomeric G4 ligands occupy a unique position in regulating tumor immunity, warranting more comprehensive and in-depth exploration.

### 4.3. Oncogene Expression Modulating G4 Ligands

While current evidence regarding the immunomodulatory effects of G4 ligands targeting oncogenes remains somewhat limited, the ability of G4 ligands to inhibit oncogene expression is well-established. Oncogenes such as *KRAS* and *c-MYC* are integral to the regulation of immune responses within the body, and targeting these genes with G4 ligands may elicit similar immunostimulatory effects to those seen with oncogene knockout or inhibition. As such, G4 ligands that target multiple oncogenes could potentially exert potent immunostimulatory effects, impacting the TME and influencing immune responses.

#### 4.3.1. c-MYC Inhibiting G4 Ligands

**Figure 6:**
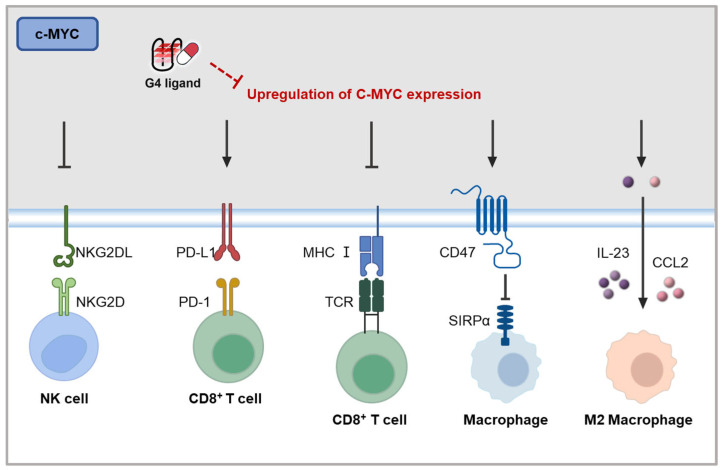
Potential mechanisms of *c-MYC* on tumor immunity [127,128,129,130,131,132].

*c-MYC* has long been recognized as a key player in regulating immune evasion. This is due to its ability to activate the transcription of various genes, including *BCL2*, which are associated with promoting tumor cell survival [125]. Research has demonstrated that *c-MYC* not only influences cell-intrinsic biology but also plays a critical role in shaping the TME [126]. Within the TME, *c-MYC* upregulates the expression of specific cytokines and their receptors, such as CCL9, CCL2, and IL-23 [127]. This upregulation activates mast cells and promotes the M2-like polarization of tumor-associated macrophages (TAMs) [128], thereby enhancing cancer cell growth and contributing to immune evasion. Additionally, *c-MYC* has been implicated in impairing the immune function of macrophages, CD4^+^ T cells, and NK cells by regulating the expression of key immunomodulatory molecules, such as PD-L1, CD47, MHC I [129,130,131], and NKG2D ligands (Figure 6) [132].

Moreover, *c-MYC* overexpression has been shown to suppress the interferon response through several mechanisms, contributing to resistance to cancer immunotherapy. It is demonstrated that *c-MYC* represses immune cell infiltration into tumors by inhibiting IFN/STING signaling in a cancer cell-intrinsic manner, particularly in TNBC cells [133].

Interestingly, research has shown that silencing or inhibiting *c-MYC* expression leads to tumor regression and TME remodeling, restoring immune responses. For instance, a study demonstrated that silencing *c-MYC* or its cofactor MAX with siRNA increased the responsiveness of melanoma cells to IFN-γ, while also enhancing the effector functions of T cells co-cultured with *MYC*-overexpressing cells [134]. Similarly, it is reported that *c-MYC* inhibition enhances CD8^+^ T cell function by suppressing Treg cell homeostasis and inhibiting the differentiation of resting Treg cells into activated Treg cells within tumors (Figure 6) [135]. Furthermore, a *c-MYC*-targeting epigenomic controller, **OTX-2002**, methylates the MYC IGD region and reduces *c-MYC* expression in hepatocellular carcinoma (HCC) models. **OTX-2002** has shown synergistic effects with anti-PD-1 therapy by reducing Treg cell numbers. In models where *c-MYC* is inactivated or inhibited, there is an increase in the expression of CCL5 and IFN I, promoting the recruitment of T cells and increasing the number of M1 macrophages [135].

Although many G4 ligands have been identified as *c-MYC* inhibitors, no studies to date have examined the immune regulatory effects of G4 ligands based on their ability to inhibit *c-MYC*. Theoretically, *c-MYC* inhibitors could have complex effects on the immune response, influencing both tumor cells and immune cells. Thus, exploring this potential in future studies is essential to fully understand the immunomodulatory effects of G4 ligands targeting *c-MYC*.

#### 4.3.2. KRAS Inhibiting G4 Ligands

The KRAS protein functions through a dynamic cycle between an inactive GDP-bound state and an active GTP-bound state, with the active form driving multiple downstream signaling pathways. In many cancers, mutations in KRAS impair GTP hydrolysis, resulting in the persistent activation of these pathways, which contributes to immune evasion [136,137,138].

**Figure 7:**
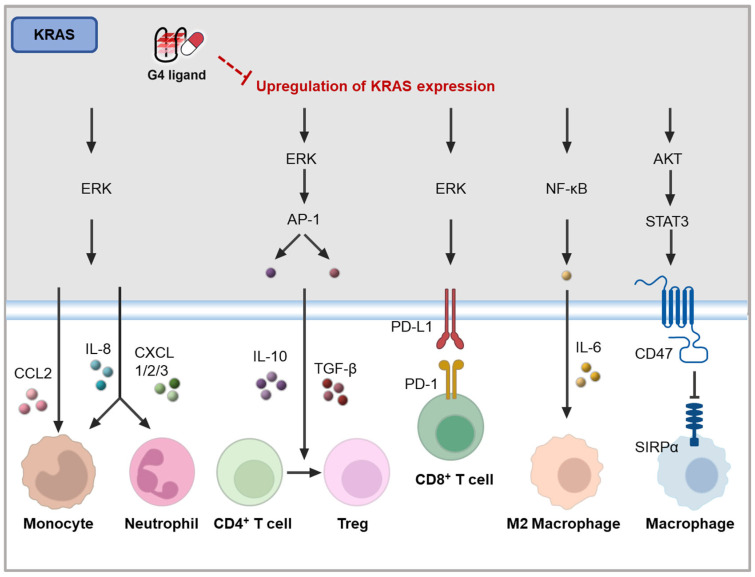
Potential mechanisms of *KRAS* on tumor immunity [139,140,141,142,149,150].

In tumor cells, mutant KRAS primarily promotes immune evasion by activating the Raf-MEK-ERK signaling pathway, which regulates the expression of various immune factors and cell surface proteins. For example, *KRAS* upregulates the expression of immune modulators, such as IL-10, TGF-β [139], and PD-L1 on cancer cells [140], leading to inhibition of T cell cytotoxicity. The secretion of IL-10 and TGF-β induced by mutant KRAS promotes the conversion of CD4^+^ T cells into Treg cells (Figure 7) [139]. Additionally, mutant KRAS stimulates the MAPK pathway, resulting in the upregulation of chemokines, like IL-8, CXCL1, CXCL2, CXCL3, and CXCL5 [141,142]. These chemokines facilitate the recruitment of suppressive immune cells, including monocytes and neutrophils, to the tumor site (Figure 7). In a mouse model of pancreatic cancer, mutant KRAS was shown to enhance GM-CSF expression via the MAPK pathway, leading to the recruitment of Gr-1^+^CD11b^+^ myeloid cells [143].

Importantly, c-MYC is an effector of the MAPK pathway activated by KRAS [144]. In tumors, mutant KRAS amplifies signaling through c-MYC, which further modulates immune responses. For instance, in lung cancer, the co-activation of KRAS and c-MYC drives the recruitment of anti-inflammatory macrophages via CCL9 and IL-23, while simultaneously excluding T cells, B cells, and natural killer (NK) cells from the tumor site [145].

Moreover, mutant KRAS contributes to immune evasion through the PI3K-AKT pathway and the NF-κB pathway. In a mouse model of *KRAS*-driven lung adenocarcinoma, CD47 was identified as a key effector of KRAS-mediated immune suppression in the TME [146]. Mechanistically, mutant KRAS activates PI3K/STAT3 signaling, which reduces the expression of miR-34a, thereby relieving its post-transcriptional repression of CD47 [146]. Furthermore, mutant KRAS activates the NF-κB pathway, which leads to the upregulation of various cytokines and chemokines, such as IL-6, which plays a role in immune suppression in KRAS-driven lung cancer (Figure 7) [147].

Additionally, KRAS modulates immune evasion by influencing the interferon response, which is also mediated by the upregulation of *c-MYC* [148]. Specifically, KRAS suppresses the expression of IRF2, which directly represses CXCL3 [141]. More recently, KRAS was found to accelerate the degradation of dsRNA in colorectal cancer, impairing dsRNA sensing and the interferon response by downregulating DExD/H-box helicase 6 (DDX60) [141]. In pancreatic ductal adenocarcinoma (PDAC), the cooperation between KRAS^G12D^ and c-MYC plays a pivotal role in regulating gene expression, particularly by repressing the IFN I pathway, thereby affecting the immune functions of NK cells, dendritic cells, and macrophages [149].

The findings suggest that targeting KRAS expression, particularly through G4 ligands, holds potential for modulating tumor immunity, but further exploration into the specific mechanisms by which these ligands could influence the immune responses associated with KRAS mutations is needed. Given the critical role KRAS plays in immune suppression within the TME, G4 ligands targeting *KRAS* may offer a novel avenue for enhancing the efficacy of immunotherapies in KRAS-driven cancers.

#### 4.3.3. Immune Genes Modulating G4 Ligands

Studies have demonstrated that G4 ligands can influence tumor immunity by upregulating the expression of genes involved in innate immune responses. A study conducted in 2021 revealed that **PDS** and **PhenDC3** could induce micronuclei formation at cell-inhibitory concentrations, leading to the activation of the cGAS-STING-IRF3 signaling pathway in the cytoplasm. This activation resulted in the increased expression of the *IFNB* gene [108]. Additionally, Seimiya’s group observed that G4 binding to splicing factor 3B subunit 2 (SF3B2) downregulated STAT1 phosphorylation and interferon-stimulated gene (ISG) expression in 3D-cultured cancer cells. Furthermore, **PhenDC3** was found to reverse the suppressive effects of G4 structures on ISG expression in vitro [151].

Given the widespread distribution of G4 sequences across various genes, stabilizing G4 structures could have significant implications for immune responses. However, the specific mechanisms underlying the interactions between G4 ligands and tumor immunity remain incompletely understood and require further exploration. This gap in knowledge presents a critical challenge for the continued development of G4-based clinical therapies, particularly for enhancing immune responses in cancer treatment. Understanding how G4 ligands modulate immune gene expression will be essential to fully harness their potential in cancer immunotherapy.

## 5. Discussion and Conclusions

This review discusses the potential of G4 ligands in tumor immunity through the following three major pathways: inducing double-stranded DNA breaks, causing telomere damage and shortening, and suppressing oncogene expression. Stabilizing G4 structures typically leads to DNA double-strand breaks, which activate the DDR pathway and trigger tumor immunity. This suggests that many G4 ligands possess immunomodulatory potential through this mechanism. However, some ligands require concentrations above toxic thresholds to effectively induce immune responses, indicating the need for further research to determine whether G4 ligands can consistently elicit tumor immunity at clinically relevant doses.

Targeting telomeric G4s is another promising strategy, as it can cause telomeric DNA damage and potentially trigger immune responses. Furthermore, these ligands may activate tumor immunity by stabilizing TERRA G4 structures or inhibiting telomerase activity, though research in these areas remains limited and warrants further investigation.

The suppression of key oncogenes, such as *c-MYC* and *KRAS*, is closely linked to cancer cell differentiation, immune cell activation, and immune escape. Theoretically, G4 ligands that selectively target oncogene G4s could inhibit oncogene expression and promote tumor immunity. However, research in this area is still in its early stages. Given the complex effects of oncogene suppression on immune responses, the role of multi-oncogene-targeting G4 ligands in tumor immunity requires more in-depth exploration.

Despite the promising potential of G4 ligands, their clinical translation remains challenging. First, the therapeutic indications and patient stratification for G4 ligands remain poorly defined. Moreover, while G4 ligands demonstrate efficacy at the biochemical and biophysical levels, they exhibit poor activity and/or selectivity when translated into cellular environments, with some ligands also showing undesirable off-target effects [152,153]. Finally, despite the rapid development of G4 ligands, their clinical translation is not aligned with the pace of their advancement due to the complexity, time constraints, and high costs involved. Further exploration of their immunotherapeutic potential may facilitate their clinical development and expand their therapeutic applications.
